# Elapsed time changes of the brain radiopharmaceutical accumulation of the amyloid PET examination using ^18^F-flutemetamol

**DOI:** 10.1007/s12149-025-02046-3

**Published:** 2025-04-19

**Authors:** Shota Takemoto, Koji Onuki, Keiko Tanimoto, Masaho Taniguchi, Takako Suero, Mio Okamoto, Satoshi Kimura, Monami Osawa, Haruka Takeshige-Amano, Noriko Nishikawa, Shigeki Aoki, Ryohei Kuwatsuru, Nobutaka Hattori, Koji Murakami

**Affiliations:** 1 Department of Radiology, Juntendo University Hospital, 3‑1‑3, Hongo, Bunkyo‑ku, Tokyo 113‑8421 Japan; 2 Department of Neurology, Juntendo University Hospital, 3‑1‑3, Hongo, Bunkyo‑ku, Tokyo 113‑8421 Japan; 3 Department of Radiology, Juntendo Tokyo Koto Geriatric Medical Center, 3‑3‑20, Shinsuna, Koto‑ku, Tokyo 136‑0075 Japan

**Keywords:** Amyloid, Brain, PET, SUVr, Centiloid scale, ^18^F-flutemetamol

## Abstract

**Objective:**

The purpose of this study was to examine how the radiopharmaceutical accumulation in the brain changes with the elapsed time between the administration of ^18^F-flutemetamol and the start of imaging, and to determine its effect on quantitative indices.

**Methods:**

The study population consisted of 25 subjects who agreed to participate in the study. After visual evaluation by the radiologist, 14 subjects tested negative for Aβ accumulation, and 11 subjects tested positive as well. The study population was treated with ^18^F-flutemeamol, and list mode acquisition was performed for 50 min starting at 60 min after the time of administration. From the acquired list data, five PET images were extracted at 10-min intervals from the start to the end of acquisition, a PET image corresponding to 20 min of acquisition from 60 min after administration, and a PET image corresponding to 20 min of acquisition from 90 min after administration, respectively. Pixel values were measured for the PET images created and quantitative indices (pixel value, SUVr, Centiloid scale) were calculated and compared.

**Results:**

In most of the PET images, pixel values showed a decreasing trend with elapsed time after radiopharmaceutical administration. Accordingly, calculated SUVr and Centiloid Scale also changed.

**Conclusions:**

Elapsed time after radiopharmaceutical administration resulted in a washout of the radiopharmaceutical accumulation in the brain. From this, it was suggested that the quantitative indices change.

## Introduction

The number of elderly people with dementia in Japan was estimated at 4.62 million in 2012 and is expected to reach approximately 7 million by 2025, or about one in five elderly people over 65 years old. Pathologic conditions that cause dementia include Alzheimer’s dementia (AD), Vascular Dementia (VaD), dementia with Lewy bodies (DLB), and frontotemporal lobar degeneration (FTLD). Also, the number of people affected by AD is the highest, accounting for the majority of all dementia cases. Although there is currently no fundamental cure, by differentiating these underlying diseases of dementia, various treatments and care are being provided for each condition. Accordingly, early detection and diagnosis of dementia as well as correct differential diagnosis of the disease are important.

According to the “Treatment of Dementia Disease Guidelines 2017”, after diagnosing suspected dementia by interview and neuropsychological examination, necessary examinations, such as imaging studies and blood and cerebrospinal fluid examinations, should be performed to identify the underlying disease of dementia [1–3]. In identifying underlying dementia, computed tomography (CT) of the head or magnetic resonance imaging (MRI) of the brain is recommended to avoid missing treatable dementia such as Chronic subdural hematoma [4–6]. Currently, amyloid imaging using Positron Emission Tomography (PET) is covered by health insurance in Japan due to the advent of the world's first treatment for AD. Therefore, the number of patients undergoing examinations and facilities conducting examinations is increasing. The usefulness of amyloid PET imaging with PET in the diagnosis of AD is also indicated in the same guideline (recommendation grade A). Amyloid PET imaging is a diagnostic imaging technique that visualizes the accumulation of amyloid-β protein (Aβ) in the brain which is thought to be a causative agent of AD using a PET tracer (such as ^18^F-flutemetamol) that has an affinity for Aβ. Currently, the evaluation of amyloid PET imaging involves the visual evaluation of appropriately luminance-adjusted PET images. For example, in ^18^F-flutemetamol, the pons luminance is adjusted to 90% of the upper limit of the image display scale and if the luminance in gray matter in the frontal lobe and posterior cingulate gyrus (i.e., the degree of accumulation of the PET tracer) exceeds a certain level, it is judged Aβ accumulation positive and the possibility of AD is indicated [7]. However, there are a small number of cases with Aβ accumulation in the borderline between positive and negative. For example, the risk management plan (RMP) for ^18^F-flutemetamol (product name: Vizamyl Intravenous Injection) lists “false negative and false positive” as a significant potential risk [8].

In other words, the evaluation of Aβ accumulation by amyloid PET imaging is largely based on the experience and subjectivity of the radiologist. Therefore, more objective evaluation methods are needed to make accurate diagnoses. The following objective methods are proposed:

1. A method for calculating the ratio of radiopharmaceutical accumulation in a reference region (e.g., cerebellum) and in an evaluation region (e.g., frontal gray matter): standardized uptake value ratio (SUVr) [9].
2. A method to evaluate the difference in SUVr for different radiopharmaceuticals using a conversion formula and using certain criteria: Centiloid scale (CL scale) [10].

We will focus on the package insert for Vizamyl intravenous injection as well as standard protocols for brain PET imaging with amyloid imaging agents. The report states that “imaging should be started after a waiting time of 60 to 120 min after radiopharmaceutical administration” [11] for amyloid PET examinations using ^18^F-flutemetamol, with a wide range of waiting time after the administration. On the other hand, PET examinations using ^18^F-Fludeoxyglucose (FDG) in Japan generally follow a protocol of a 60-min waiting time after radiopharmaceutical administration. Accordingly, considering the efficiency of the patient's waiting time and examination schedule a 60 min wait after administration is desirable for amyloid PET examinations using ^18^F-flutemetamol as well. Also, long waiting time are a great mental burden for AD patients, so it is desirable to keep waiting time as short as possible. However, for the calculation of the CL scale, quantitative indices, the standard examination protocol [12] is to wait 90 min after the radiopharmaceutical administration, a factor that causes difficulties in setting the waiting time.

Changes in waiting time after radiopharmaceutical administration and in the amount of radiopharmaceutical accumulation in the brain have been reported in amyloid PET scans using ^18^F-flutemetamol [13]. However, no clear findings have been made and the effects on quantitative indices, such as SUVr and CL scale, are not clear. If the quantitative indices determined from the amount of radiopharmaceutical accumulation in the brain vary with the waiting time, then using a uniform standard or threshold for evaluation could lead to misdiagnosis.

The purpose of this study was to examine the changes in radiopharmaceutical accumulation in the brain over the elapsed time between the administration of ^18^F-flutemetamol and the start of acquisition, and to determine the effect of these changes on quantitative indices.

## Materials and methods

### Subjects and equipment used

Twenty five subjects who agreed to the study content were included in this study. An amyloid PET scan was performed using ^18^F-flutemetamol as the radiopharmaceutical, and visual evaluation by the radiologist revealed that Aβ accumulation was negative in 14 subjects [twelve males and two females, aged 48–80 years (mean ± SD, 69.4 ± 8.4)] and positive in 11 subjects [one male and ten females, aged 59–84 years (mean ± SD, 75.6 ± 6.4)].

The PET/CT system used in this study was a Celesteion PCA- 9000 A/3 A (CANON MEDICAL SYSTEMS CORPORATION, Tochigi, Japan). The imaging conditions were those used in our clinical practice. Details are TOF 3D OSEM (Iterations:4, Subsets:10), no PSF correction, Gaussian filter (FWHM:4 mm), pixel size 2.03875 mm.

### Collection methods and image creation

**Fig. 1:**
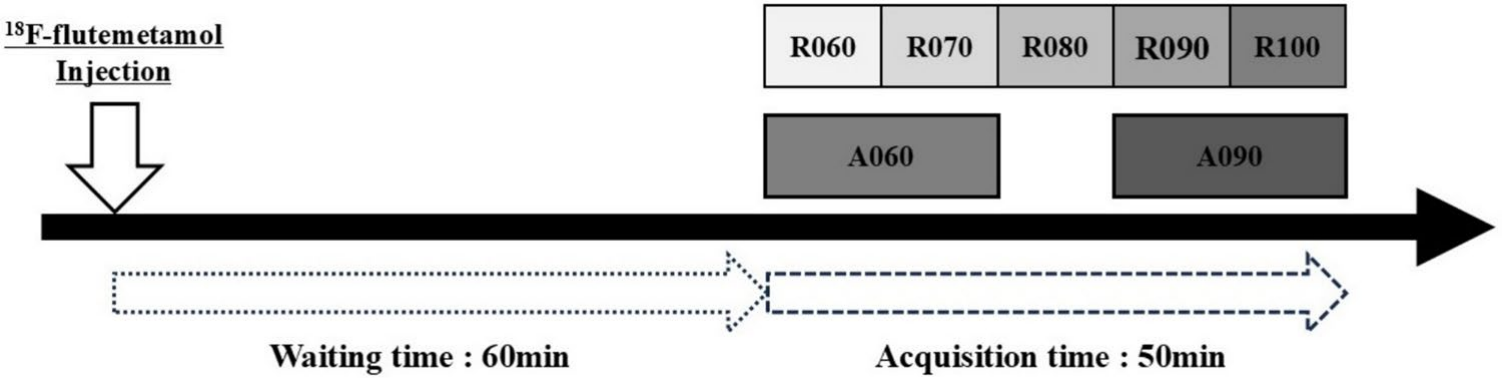
Collection method

Starting with the administration of the radiopharmaceutical to the subject, 50-min list mode acquisition was performed starting 60 min later. From the obtained 50-min list data, data were extracted using the following conditions, and multiple PET images were generated for each subject (Fig. 1).

1. PET images segmented at 10-min intervals from the start of imaging to the end of imaging (R060, R070, R080, R090, R100).
2. PET images corresponding to 20 min acquisition at 60 to 80 min after radiopharmaceutical administration (A060).
3. PET images corresponding to 20 min acquisition at 90 to 110 min after radiopharmaceutical administration (A090).

### Analysis and evaluation

Quantitative indices were calculated by measuring pixel values of multiple PET images created using the analysis application “VIZCalc” in the “nuclear medicine image analysis software medi + FALCON” (Nippon Medi-Physics Co., Ltd, Tokyo, Japan). In this study, the SUVr and CL scale calculated from the average pixel value and the average pixel value in the region of interest were used for evaluation.

### Time changes and quantitative indices

**Fig. 2:**
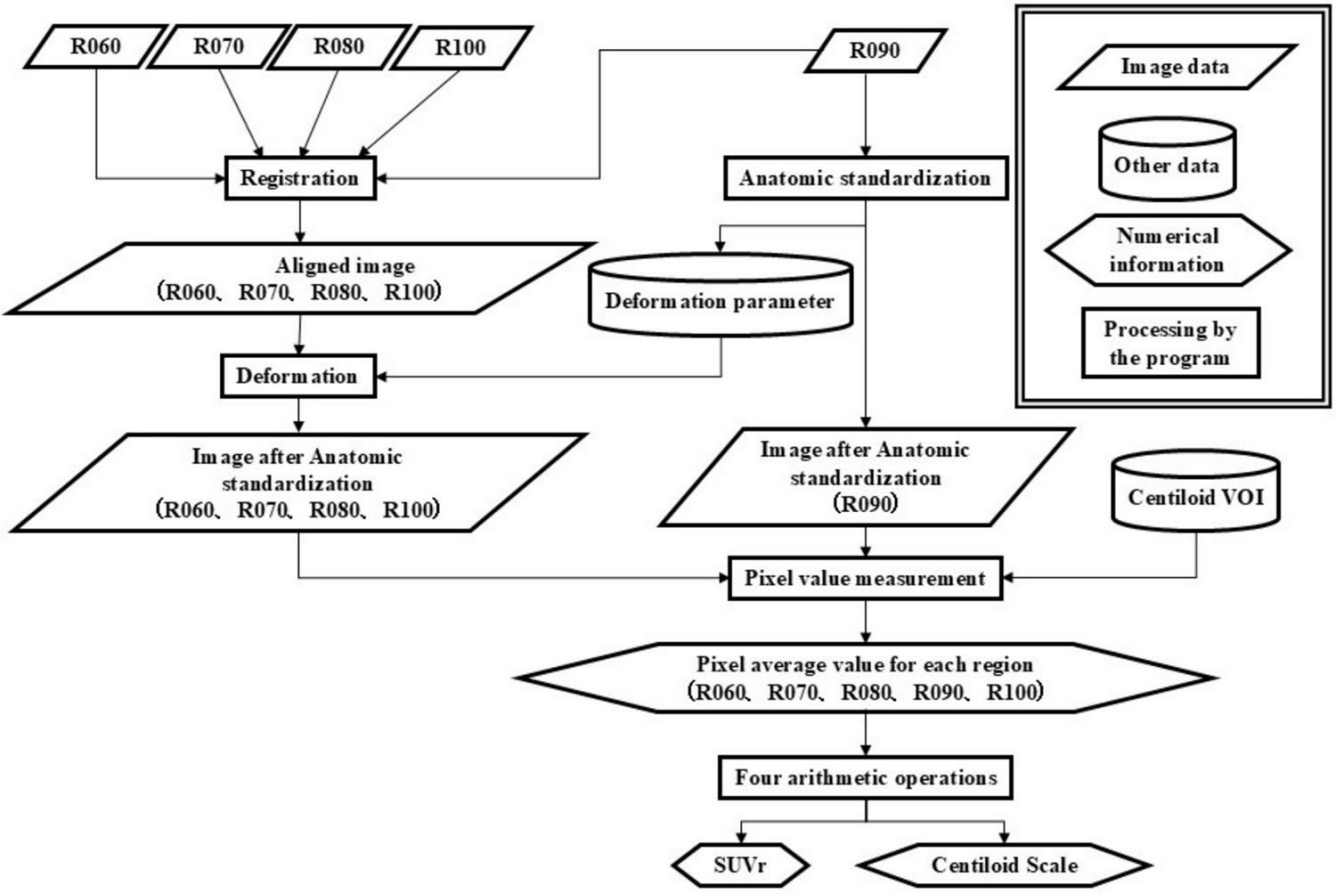
Anatomic standardization using VIZCalc

First, the relationship between elapsed time since radiopharmaceutical administration and pixel values and quantitative indices was examined. PET images (R090) corresponding to 90 min of waiting time were subjected to anatomic standardization [12, 15] using VIZCalc. To reduce errors in anatomic standardization due to differences in images, the other PET images (R060, R070, R080, and R100) were aligned with standard to R090. Afterward, the deformation parameters obtained from the anatomic standardization of R090 were applied to the other anatomic standardization (Fig. 2).

**Fig. 3:**
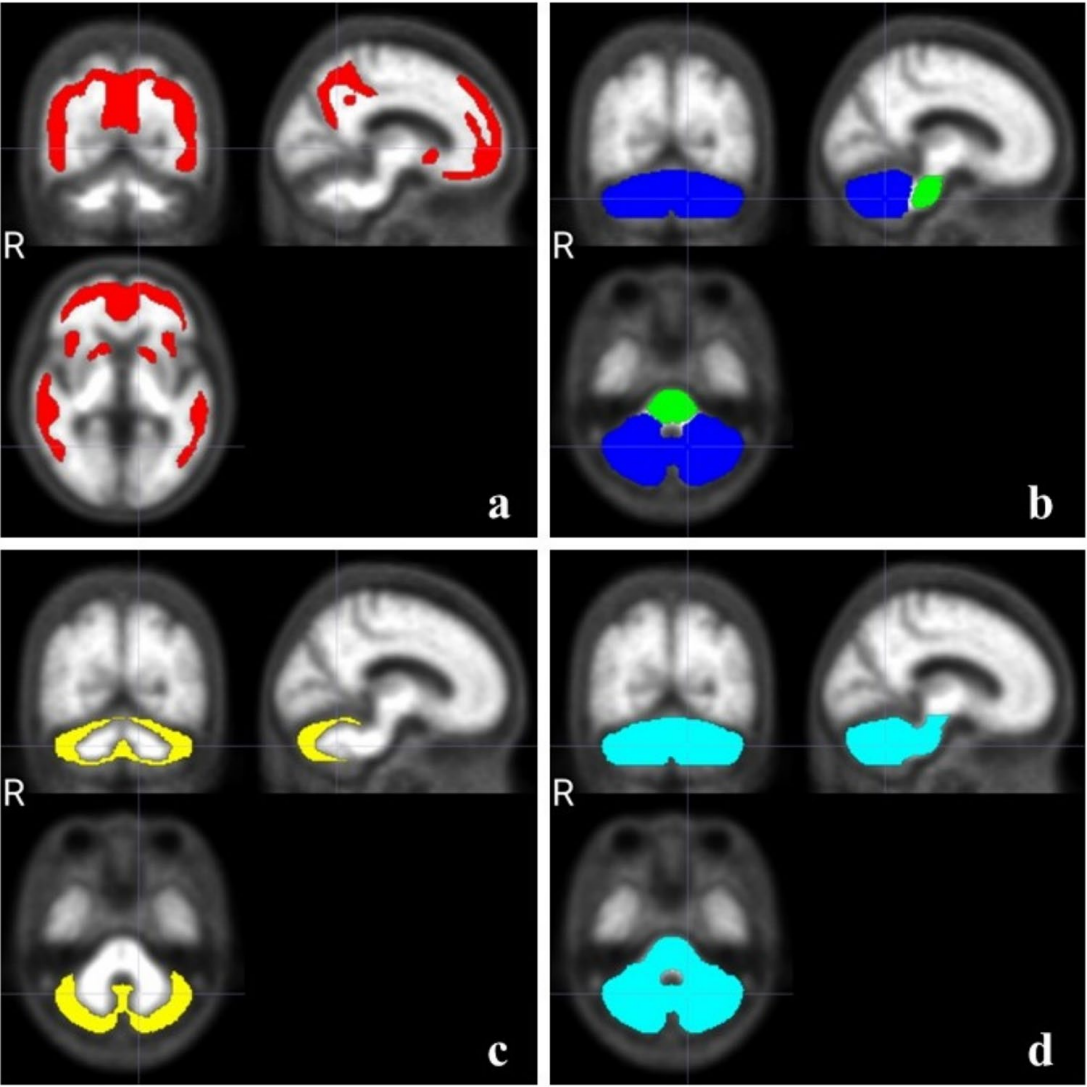
Region of interest for CL calculation built into VIZCalc (**a** Cortex, **b** WC, Pons, **c** CG, **d** WC + B)

PET images after anatomic standardization were measured for pixel values using the region of interest (CL VOI) [10, 12] for CL calculation, which is installed by VIZCalc (Tables 1, 2). CL VOI with the five regions of Cerebral Cortex (Cortex), Whole Cerebellum (WC), Pons (Pons), Cerebellar Gray (CG) and Whole Cerebellum plus Brainstem (WC+B) (Fig. 3).

**Table 1:** Mean pixel value in each region of the negative group

Negative	列2	Pixel value (mean)				
No.	Region	R060	R070	R080	R090	R100
001	Cortex	6287.01	5570.07	4992.12	4484.54	4196.59
	WC	5937.36	5305.11	4772.00	4355.25	3992.20
	Pons	9696.91	8921.06	8143.14	7660.84	7069.04
	CG	4808.20	4155.18	3619.63	3248.87	2910.68
	WC + B	6498.27	5851.63	5286.09	4861.85	4466.90
002	Cortex	4136.22	3726.50	3418.22	3129.48	2950.27
	WC	4100.53	3727.56	3415.16	3170.23	2962.14
	Pons	7118.58	6522.57	6014.83	5565.19	5201.60
	CG	3213.55	2838.64	2547.81	2319.86	2145.32
	WC + B	4565.34	4157.95	3816.18	3540.51	3315.37
003	Cortex	5492.42	4847.99	4402.69	4000.57	3679.12
	WC	5219.94	4637.68	4190.85	3854.94	3531.08
	Pons	8765.44	7932.39	7393.03	6722.65	6474.21
	CG	4224.08	3630.10	3165.02	2848.49	2535.84
	WC + B	5757.38	5136.60	4679.42	4296.18	3981.82
004	Cortex	5503.25	4953.23	4540.47	4223.98	4002.37
	WC	5012.90	4562.61	4204.67	3916.53	3639.41
	Pons	8063.95	7531.99	7054.29	6705.54	6458.50
	CG	4133.41	3670.90	3294.82	2976.06	2711.82
	WC + B	5482.71	5020.30	4651.67	4351.04	4076.20
005	Cortex	3869.41	3457.58	3113.08	2847.25	2659.16
	WC	3682.19	3278.24	2980.88	2738.37	2520.28
	Pons	6176.95	5729.54	5362.31	5016.64	4669.25
	CG	2879.29	2484.03	2191.95	1960.80	1759.59
	WC + B	4067.49	3654.64	3351.02	3093.55	2853.58
006	Cortex	8016.94	7070.75	6229.77	5569.55	5064.54
	WC	7153.79	6303.69	5552.34	4993.68	4482.52
	Pons	11,422.50	10,905.90	9849.12	9180.11	8656.98
	CG	5811.58	4859.54	4160.62	3628.69	3174.25
	WC + B	7815.15	7014.58	6224.86	5647.89	5142.55
007	Cortex	4518.65	4077.03	3699.05	3418.06	3171.43
	WC	4119.30	3638.79	3271.83	2971.76	2780.23
	Pons	6992.17	6369.68	5847.39	5399.17	5086.63
	CG	3187.66	2743.52	2396.83	2147.11	1985.57
	WC + B	4559.60	4062.07	3673.69	3352.05	3141.99
008	Cortex	4820.26	4247.49	3825.84	3519.76	3209.83
	WC	5014.28	4451.08	4010.85	3673.76	3382.70
	Pons	8344.95	7636.62	7144.26	6823.33	6279.33
	CG	3891.58	3360.94	2915.62	2588.86	2393.19
	WC + B	5534.18	4954.46	4505.31	4167.91	3837.92
009	Cortex	3024.52	2646.17	2363.05	2143.36	1971.51
	WC	3388.44	3052.89	2781.31	2554.05	2304.37
	Pons	5792.81	5310.93	5000.18	4508.19	4344.93
	CG	2556.90	2248.06	1999.41	1801.51	1572.92
	WC + B	3758.85	3405.47	3130.42	2864.77	2623.95
010	Cortex	4532.66	4029.13	3639.02	3376.70	3112.51
	WC	4159.12	3769.19	3430.95	3220.59	2974.48
	Pons	7280.18	6691.90	6261.56	5803.99	5354.24
	CG	3146.96	2808.37	2495.82	2286.80	2080.32
	WC + B	4623.89	4212.59	3858.06	3613.13	3343.11
011	Cortex	3910.42	3449.99	3115.34	2879.85	2640.89
	WC	4391.61	3915.51	3563.57	3281.24	3041.95
	Pons	7613.50	7117.12	6615.33	6316.96	5844.54
	CG	3392.22	2914.50	2592.83	2345.79	2122.76
	WC + B	4897.83	4417.34	4048.58	3761.48	3489.51
012	Cortex	5499.18	4801.76	4207.66	3798.46	3491.73
	WC	5482.40	4805.69	4172.16	3764.77	3405.54
	Pons	8642.24	7777.17	6879.76	6561.36	6093.80
	CG	4401.45	3756.49	3168.76	2819.60	2501.23
	WC + B	5972.24	5276.93	4607.74	4210.34	3836.55
013	Cortex	4932.87	4379.24	3963.16	3595.93	3264.26
	WC	5121.86	4539.29	4082.02	3729.42	3474.64
	Pons	8388.27	7694.15	7211.64	6795.18	6172.49
	CG	4120.75	3559.52	3106.03	2747.18	2548.80
	WC + B	5615.24	5022.78	4558.02	4202.86	3895.14
014	Cortex	3263.46	2879.49	2604.07	2378.23	2190.55
	WC	3062.50	2737.81	2493.64	2297.54	2101.48
	Pons	5488.97	5126.42	4720.00	4269.43	3979.05
	CG	2346.31	2003.86	1794.50	1619.52	1462.71
	WC + B	3439.85	3107.76	2840.73	2610.89	2397.16

**Table 2:** Mean pixel value in each region of the positive group

Positive	列2	Pixel value (mean)				
No.	Region	R060	R070	R080	R090	R100
001	Cortex	8284.80	7473.83	6833.34	6369.64	5980.70
	WC	6987.47	6165.56	5644.31	5176.47	4689.56
	Pons	11,581.40	10,373.40	9456.67	8905.43	8133.95
	CG	5616.28	4831.24	4367.13	3942.78	3630.11
	WC + B	7699.98	6817.53	6239.51	5763.18	5233.73
002	Cortex	6129.27	5709.30	5222.29	4957.16	4622.51
	WC	4585.41	4099.29	3749.70	3479.31	3242.97
	Pons	7816.52	6996.88	6364.98	6033.82	5492.40
	CG	3543.50	3150.22	2852.92	2594.91	2416.26
	WC + B	5077.35	4548.53	4158.25	3882.00	3603.07
003	Cortex	11,052.40	10,403.70	9876.25	9392.92	8899.53
	WC	7349.82	6674.93	6114.28	5785.34	5376.03
	Pons	12,368.20	11,370.90	10,576.90	9898.66	9261.21
	CG	5744.29	5150.21	4652.67	4296.72	3936.94
	WC + B	8122.83	7403.56	6808.44	6432.30	5984.72
004	Cortex	5666.05	5058.11	4583.70	4176.23	3860.65
	WC	5032.68	4502.38	3990.48	3646.32	3314.24
	Pons	8010.16	7240.97	6718.76	6102.24	5633.17
	CG	3971.83	3426.73	2949.81	2602.09	2317.31
	WC + B	5493.90	4932.76	4418.19	4036.19	3681.05
005	Cortex	13,563.10	12,648.30	11,733.40	11,056.40	10,434.00
	WC	9439.73	8334.89	7405.33	6880.20	6231.00
	Pons	15,739.30	14,354.70	12,997.40	12,177.10	11,540.50
	CG	7685.81	6602.32	5714.44	5189.58	4581.49
	WC + B	10,369.20	9237.29	8250.43	7698.13	7044.47
006	Cortex	7256.05	6479.08	5916.32	5319.47	4844.98
	WC	6481.31	5684.38	5073.51	4597.88	4148.04
	Pons	10,066.10	9334.79	8466.66	7797.61	7124.90
	CG	5087.63	4233.01	3690.67	3292.66	2909.44
	WC + B	7073.23	6272.02	5623.38	5122.96	4633.22
007	Cortex	4841.99	4399.46	4034.37	3692.42	3457.41
	WC	4560.74	4048.54	3643.27	3396.50	3051.59
	Pons	7514.46	6904.60	6272.25	6093.85	5688.77
	CG	3598.51	3070.23	2698.66	2415.71	2119.98
	WC + B	5009.10	4483.19	4052.11	3815.84	3455.97
008	Cortex	7415.19	6869.51	6419.38	6048.45	5394.91
	WC	5188.37	4700.81	4443.43	4064.86	3558.66
	Pons	7826.49	7358.13	6933.01	6331.27	5238.60
	CG	4418.79	3855.22	3621.51	3280.80	3026.63
	WC + B	5612.02	5121.77	4841.63	4430.18	3847.71
009	Cortex	7610.75	7022.87	6448.22	6032.79	5719.49
	WC	4503.19	4097.38	3731.29	3428.45	3155.87
	Pons	7173.89	6826.10	6219.05	5950.55	5461.84
	CG	3726.08	3255.07	2901.39	2583.12	2346.45
	WC + B	4904.42	4509.99	4111.64	3814.67	3511.52
010	Cortex	7759.69	6867.27	6125.92	5508.15	5105.26
	WC	6510.69	5628.87	4990.11	4386.31	3969.57
	Pons	10,838.00	9646.89	8610.44	7888.69	7250.45
	CG	5067.67	4246.58	3687.79	3106.51	2774.79
	WC + B	7192.19	6264.60	5569.84	4941.11	4490.31
011	Cortex	8458.84	7591.96	7044.00	6522.05	6069.20
	WC	6101.56	5355.05	4868.41	4455.15	4081.64
	Pons	10,107.60	9268.57	8477.42	8070.92	7359.84
	CG	4918.50	4188.43	3665.68	3300.58	2976.66
	WC + B	6707.82	5948.60	5419.44	5011.50	4589.80

SUVr (Cerebral Cortex/reference parts) was obtained from the average pixel value for each measured region (Tables 3, 4). The relationship between the measured average pixel values and SUVr and the elapsed time since the radiopharmaceutical was injected was verified.

**Table 3:** SUVr of each region in the negative group by elapsed time

Negative	列2	SUVr				
No.	Regions	R060	R070	R080	R090	R100
001	WC	1.059	1.050	1.046	1.030	1.051
	Pons	0.648	0.624	0.613	0.585	0.594
	CG	1.308	1.341	1.379	1.380	1.442
	WC + B	0.967	0.952	0.944	0.922	0.939
002	WC	1.009	1.000	1.001	0.987	0.996
	Pons	0.581	0.571	0.568	0.562	0.567
	CG	1.287	1.313	1.342	1.349	1.375
	WC + B	0.906	0.896	0.896	0.884	0.890
003	WC	1.052	1.045	1.051	1.038	1.042
	Pons	0.627	0.611	0.596	0.595	0.568
	CG	1.300	1.335	1.391	1.404	1.451
	WC + B	0.954	0.944	0.941	0.931	0.924
004	WC	1.098	1.086	1.080	1.079	1.100
	Pons	0.682	0.658	0.644	0.630	0.620
	CG	1.331	1.349	1.378	1.419	1.476
	WC + B	1.004	0.987	0.976	0.971	0.982
005	WC	1.051	1.055	1.044	1.040	1.055
	Pons	0.626	0.603	0.581	0.568	0.570
	CG	1.344	1.392	1.420	1.452	1.511
	WC + B	0.951	0.946	0.929	0.920	0.932
006	WC	1.121	1.122	1.122	1.115	1.130
	Pons	0.702	0.648	0.633	0.607	0.585
	CG	1.379	1.455	1.497	1.535	1.596
	WC + B	1.026	1.008	1.001	0.986	0.985
007	WC	1.097	1.120	1.131	1.150	1.141
	Pons	0.646	0.640	0.633	0.633	0.623
	CG	1.418	1.486	1.543	1.592	1.597
	WC + B	0.991	1.004	1.007	1.020	1.009
008	WC	0.961	0.954	0.954	0.958	0.949
	Pons	0.578	0.556	0.536	0.516	0.511
	CG	1.239	1.264	1.312	1.360	1.341
	WC + B	0.871	0.857	0.849	0.844	0.836
009	WC	0.893	0.867	0.850	0.839	0.856
	Pons	0.522	0.498	0.473	0.475	0.454
	CG	1.183	1.177	1.182	1.190	1.253
	WC + B	0.805	0.777	0.755	0.748	0.751
010	WC	1.090	1.069	1.061	1.048	1.046
	Pons	0.623	0.602	0.581	0.582	0.581
	CG	1.440	1.435	1.458	1.477	1.496
	WC + B	0.980	0.956	0.943	0.935	0.931
011	WC	0.890	0.881	0.874	0.878	0.868
	Pons	0.514	0.485	0.471	0.456	0.452
	CG	1.153	1.184	1.202	1.228	1.244
	WC + B	0.798	0.781	0.769	0.766	0.757
012	WC	1.003	0.999	1.009	1.009	1.025
	Pons	0.636	0.617	0.612	0.579	0.573
	CG	1.249	1.278	1.328	1.347	1.396
	WC + B	0.921	0.910	0.913	0.902	0.910
013	WC	0.963	0.965	0.971	0.964	0.939
	Pons	0.588	0.569	0.550	0.529	0.529
	CG	1.197	1.230	1.276	1.309	1.281
	WC + B	0.878	0.872	0.869	0.856	0.838
014	WC	1.066	1.052	1.044	1.035	1.042
	Pons	0.595	0.562	0.552	0.557	0.551
	CG	1.391	1.437	1.451	1.468	1.498
	WC + B	0.949	0.927	0.917	0.911	0.914

**Table 4:** SUVr of each region in the positive group by elapsed time

Positive	列2	SUVr				
No.	Regions	R060	R070	R080	R090	R100
001	WC	1.186	1.212	1.211	1.230	1.275
	Pons	0.715	0.720	0.723	0.715	0.735
	CG	1.475	1.547	1.565	1.616	1.648
	WC + B	1.076	1.096	1.095	1.105	1.143
002	WC	1.337	1.393	1.393	1.425	1.425
	Pons	0.784	0.816	0.820	0.822	0.842
	CG	1.730	1.812	1.831	1.910	1.913
	WC + B	1.207	1.255	1.256	1.277	1.283
003	WC	1.504	1.559	1.615	1.624	1.655
	Pons	0.894	0.915	0.934	0.949	0.961
	CG	1.924	2.020	2.123	2.186	2.261
	WC + B	1.361	1.405	1.451	1.460	1.487
004	WC	1.126	1.123	1.149	1.145	1.165
	Pons	0.707	0.699	0.682	0.684	0.685
	CG	1.427	1.476	1.554	1.605	1.666
	WC + B	1.031	1.025	1.037	1.035	1.049
005	WC	1.437	1.518	1.584	1.607	1.675
	Pons	0.862	0.881	0.903	0.908	0.904
	CG	1.765	1.916	2.053	2.130	2.277
	WC + B	1.308	1.369	1.422	1.436	1.481
006	WC	1.120	1.140	1.166	1.157	1.168
	Pons	0.721	0.694	0.699	0.682	0.680
	CG	1.426	1.531	1.603	1.616	1.665
	WC + B	1.026	1.033	1.052	1.038	1.046
007	WC	1.062	1.087	1.107	1.087	1.133
	Pons	0.644	0.637	0.643	0.606	0.608
	CG	1.346	1.433	1.495	1.529	1.631
	WC + B	0.967	0.981	0.996	0.968	1.000
008	WC	1.429	1.461	1.445	1.488	1.516
	Pons	0.947	0.934	0.926	0.955	1.030
	CG	1.678	1.782	1.773	1.844	1.782
	WC + B	1.321	1.341	1.326	1.365	1.402
009	WC	1.690	1.714	1.728	1.760	1.812
	Pons	1.061	1.029	1.037	1.014	1.047
	CG	2.043	2.158	2.222	2.335	2.438
	WC + B	1.552	1.557	1.568	1.581	1.629
010	WC	1.192	1.220	1.228	1.256	1.286
	Pons	0.716	0.712	0.711	0.698	0.704
	CG	1.531	1.617	1.661	1.773	1.840
	WC + B	1.079	1.096	1.100	1.115	1.137
011	WC	1.386	1.418	1.447	1.464	1.487
	Pons	0.837	0.819	0.831	0.808	0.825
	CG	1.720	1.813	1.922	1.976	2.039
	WC + B	1.261	1.276	1.300	1.301	1.322

All pixel values are decay corrected to correspond to 60 min after administration.

### CL scale in clinical acquisition conditions

Next, we verified the effect of waiting time after radiopharmaceutical administration on the CL scale for PET images corresponding to a 20-min acquisition, which is performed in our clinical examination. PET images (A060) corresponding to a 20-min acquisition time from 60 min of waiting time and PET images (A090) corresponding to a 20-min acquisition time from 90 min of waiting time are covered. For the two PET images (A060 and A090), the CL scale was calculated using VIZCalc to verify the effect of the difference in waiting time. The reported conversion formula to calculate the CL scale is given below (Table 5) [10, 12, 14]:

**Table 5:** Summary statistics of CL scale for A060 and A090

列1	Centiloid	
No.	A060	A090
N- 001	1.982	0.385
N- 002	− 2.072	− 5.142
N- 003	3.824	0.508
N- 004	6.281	7.509
N- 005	8.615	1.859
N- 006	12.177	10.457
N- 007	13.282	14.142
N- 008	− 5.634	− 9.687
N- 009	− 17.303	− 23.198
N- 010	7.141	1.982
N- 011	− 15.092	− 19.514
N- 012	− 0.598	− 2.317
N- 013	− 3.546	− 9.319
N- 014	6.895	1.368
P- 001	23.968	27.039
P- 002	42.393	48.166
P- 003	64.993	74.820
P- 004	16.107	13.651
P- 005	58.975	74.697
P- 006	15.739	15.247
P- 007	11.194	9.229
P- 008	47.797	57.747
P- 009	89.928	91.279
P- 010	25.934	29.127
P- 011	48.780	54.553

*N* negative, *P* positive

$$CL=122.83\times {SUV}_{rFlute}-126.13$$

The subjects were divided into a negative group (14 subjects) and a positive group (11 subjects), and changes in waiting time and CL were compared in each group. For statistical analysis, “Paired *t*-test” was performed using EZR [16].

## Results

### Pixel value change in CL VOI

**Fig. 4:**
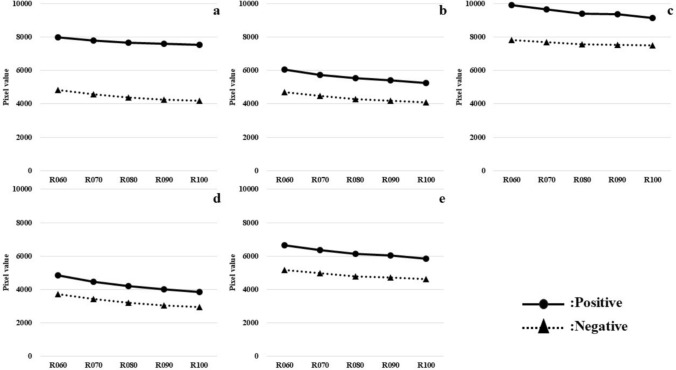
Change in pixel values with elapsed time after radiopharmaceutical in each region of interest (**a** Cortex, **b** WC, **c** Pons, **d** CG, **e** WC + B)

Changes in pixel values over elapsed time after radiopharmaceutical administration for each region of interest are shown separately for the negative and positive groups (Fig. 4). In both groups, all regions (Cortex, WC, Pons, CG, and WC + B) showed a trend of decrease gradually in pixel values with elapsed time after radiopharmaceutical administration.

**Fig. 5:**
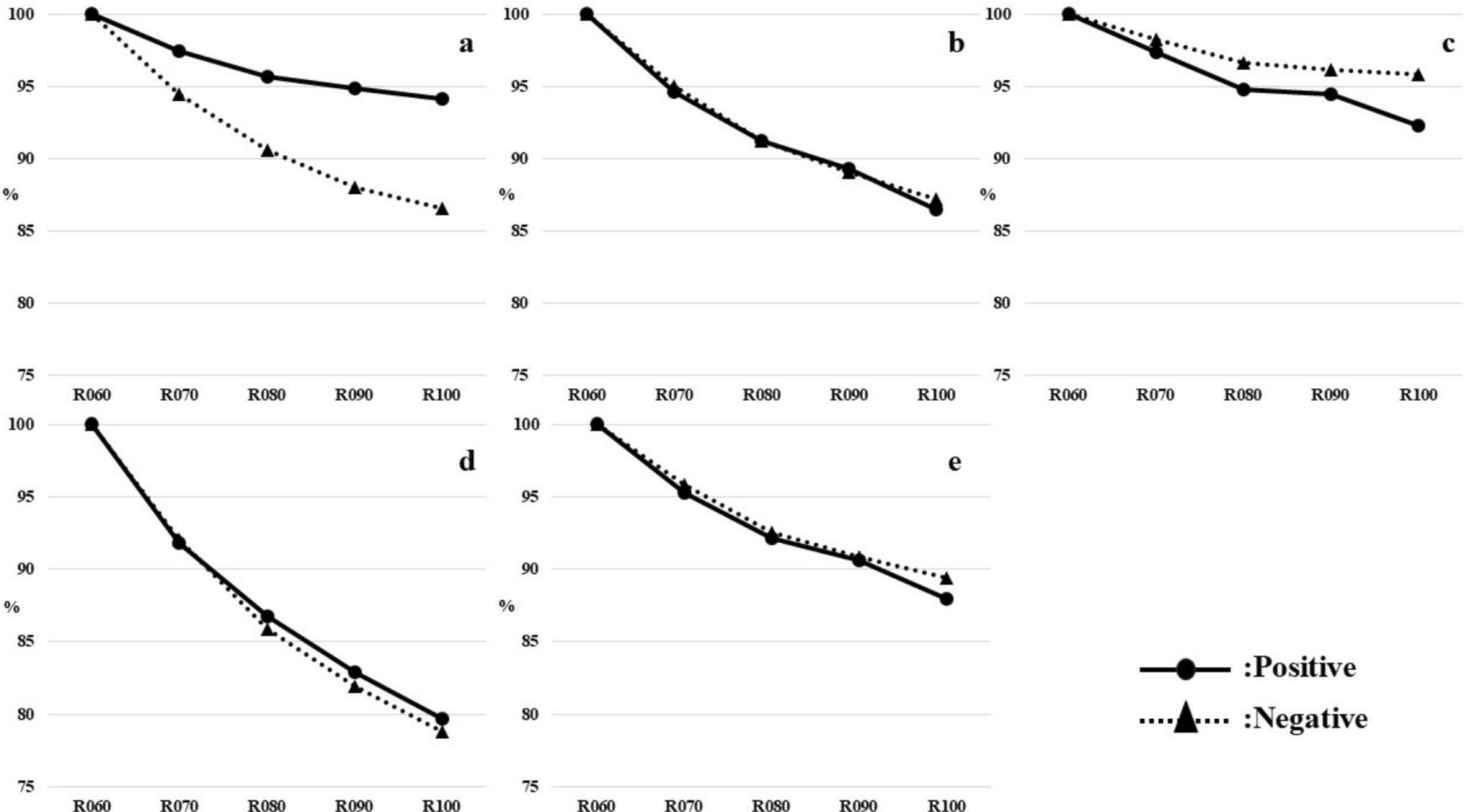
Graph of average measurements for each region converted to percentages (**a** Cortex, **b** WC, **c** Pons, **d** CG, **e** WC + B)

Also, the average measurements obtained from D060 were set at 100%. After that, the average measurement of each region by elapsed time was then converted to a percentage. As a result, the pixel values measured in the Cortex region showed a trend of decrease gradually in the positive group than in the negative group (Fig. 5).

About WC, Pons, CG, and WC + B regions, not much difference was observed. However, comparing the Pons and CG regions to the Pons region showed a decrease gradually in pixel values.

### Change in SUVr

**Fig. 6:**
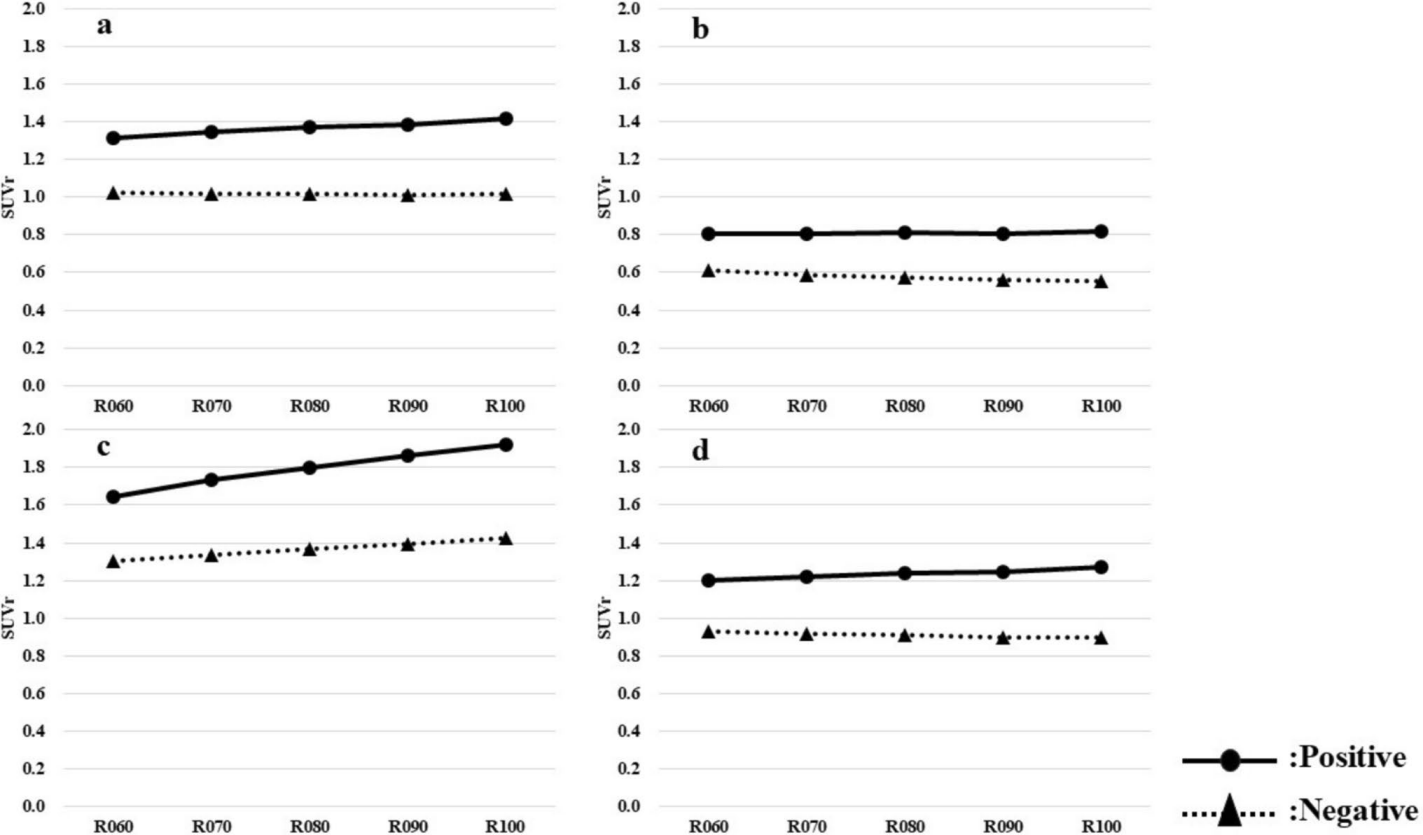
The changes in SUVr calculated using each region as reference parts with elapsed time after radiopharmaceutical administration (**a** WC, **b** Pons, **c** CG, **d** WC + B)

The changes in SUVr were calculated using the WC, Pons, CG, and WC + B regions as reference parts with elapsed time after radiopharmaceutical administration (Fig. 6). It was found that the trend of change with elapsed time after radiopharmaceutical administration differed depending on the reference parts at the time of SUVr calculation.

When the WC region was used as the reference, there was an increasing trend in the positive group, while no change was shown for the negative group.

When the Pons region was used as the reference, no change was shown for the positive group, but there was a decreasing trend in the negative group.

When the CG region was used as the reference, there was an increasing trend in both positive and negative groups.

When the WC + B region was used as the reference, the positive group showed a slight increasing trend, while the negative group showed a slight decreasing trend.

### Changes in CL scale and SUVr under clinical collection conditions

**Fig. 7:**
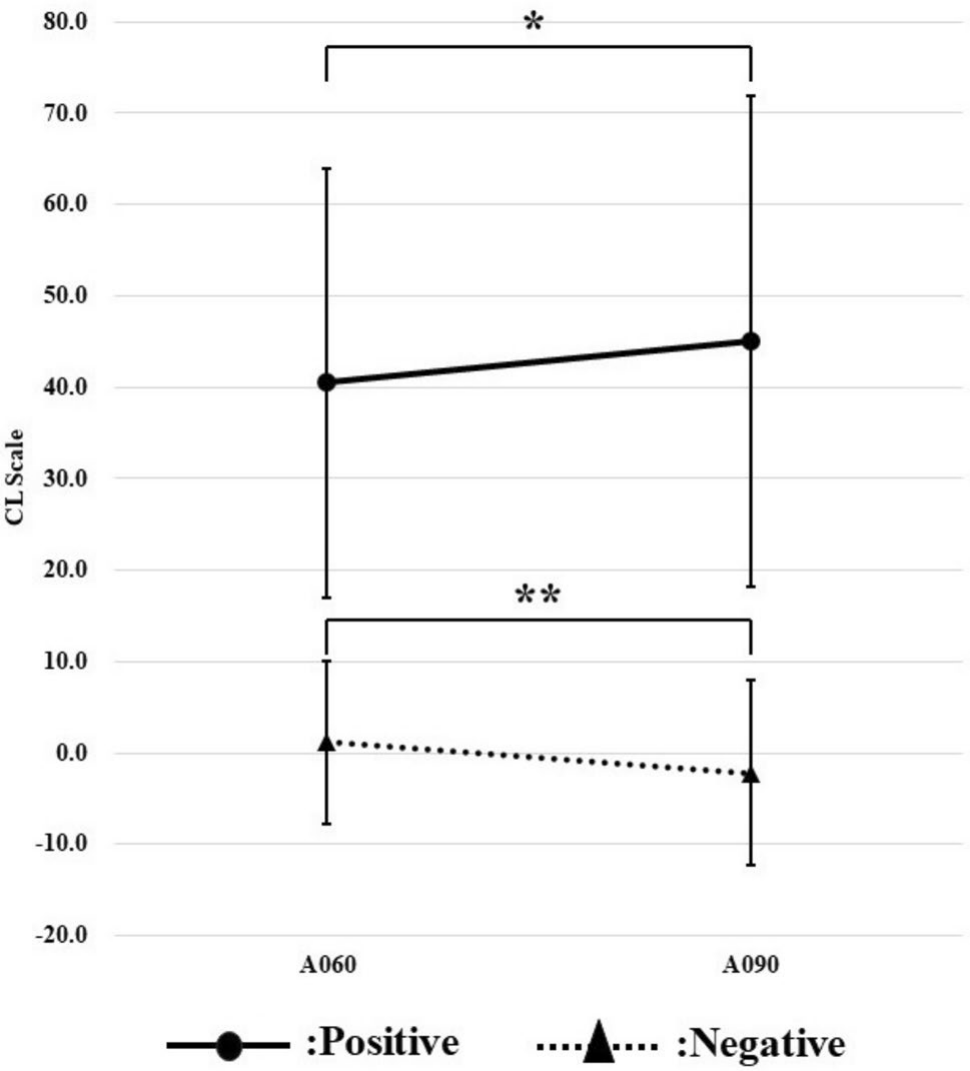
Comparison of the CL scale calculated by our clinical examination conditions with the CL scale calculated by the standard protocol in the Centiloid Project

We examined a comparison between the protocol used in our clinical examination (60 min waiting time and 20 min acquisition time) and the standard protocol of the Centroid Project (90 min waiting time and 20 min acquisition time) (Fig. 7). CL scale is calculated from the SUVr of the WC region reference. Therefore, similar to the SUVr of the WC region reference in the results of Fig. 6, the positive group showed an increasing trend with elapsed time, while the negative group showed a decreasing trend. The calculated CL scale showed a significant difference between the positive and negative groups due to differences in waiting time.

**Fig. 8:**
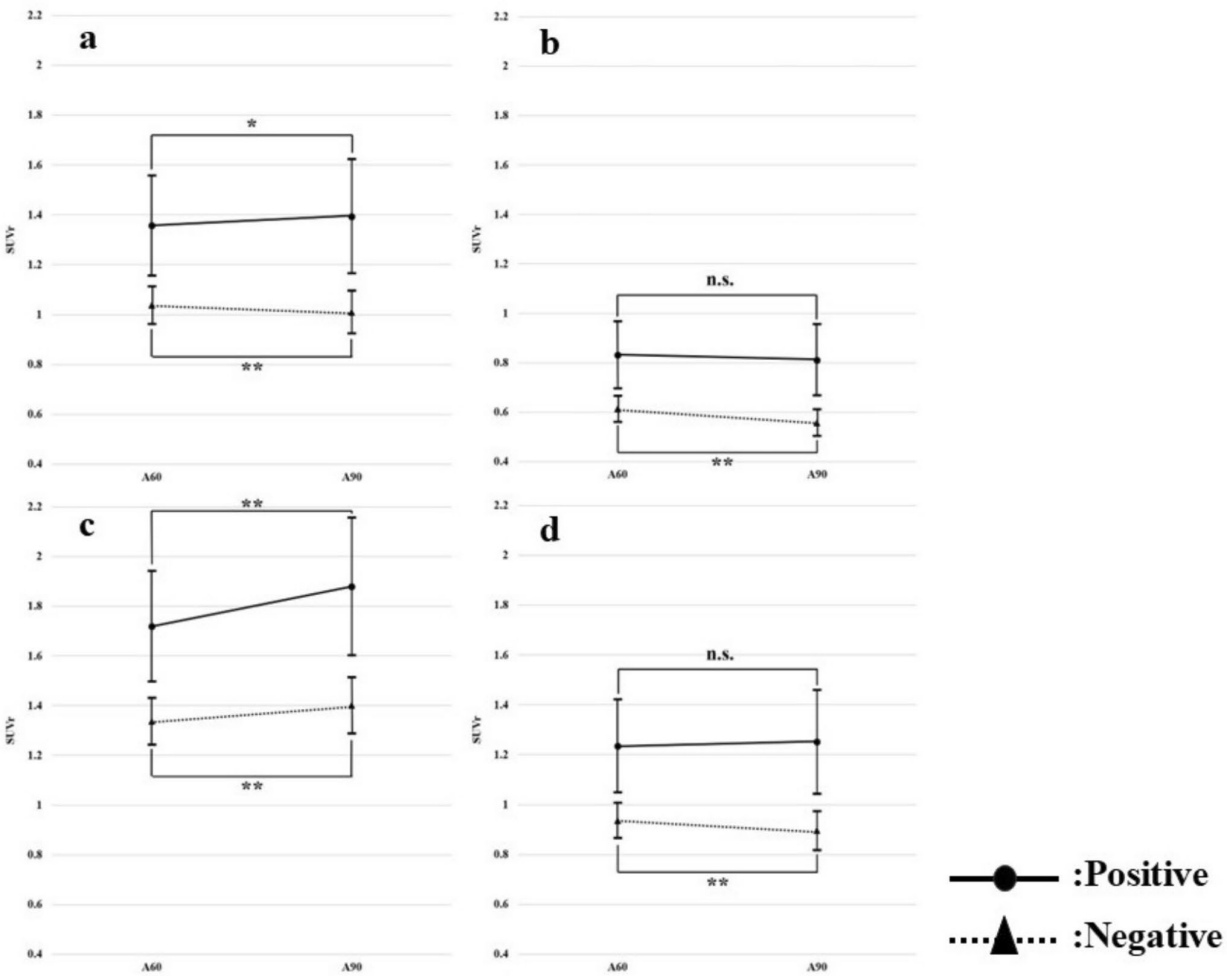
Comparison of SUVr by each region reference with waiting times of 60 and 90 min (**a** WC, **b** Pons, **c** CG, **d** WC + B)

In addition, the comparison of waiting times of 60 min and 90 min is also shown for the SUVr of each reference region (Fig. 8). From this, the CL scale is seen to show a tendency toward SUVr. In other regions, for example, according to the Pons region reference, the positive group showed a slight decreasing trend over elapsed time, while the negative group showed a decreasing trend. Furthermore, there was no significant difference in the positive group, but a significant difference was seen in the negative group.

## Discussion

In the results of Figs. 4 and 5, there was a decrease in pixel values with elapsed time after radiopharmaceutical administration. Therefore, it was suggested that the radiopharmaceuticals distributed in the brain tissue were washed out with elapsed time. Additionally, the trends of change in the Pons region with the entire region predominantly occupied by white matter, and in the CG region with the entire region predominantly occupied by gray matter were compared. Therefore, it can be said that white matter is slower to wash out than gray matter. In general, the white matter has less blood flow than the gray matter [17–19] and it is assumed that the speed of wash out is proportional to the cerebral blood flow. Assume that cerebral blood flow is lower in the positive group (≈ more amyloid plaque deposition in the cerebrum) than in the negative group. Accordingly, it can be said that the washout in the Cortex region of the positive group is consistent with the slower washout in the Cortex region than in the negative group.

The results of Figs. 6 and 7 showed that the quantitative indices (SUVr and CL) changed with elapsed time after radiopharmaceutical administration. In addition, the results of Fig. 8 show that the trend of SUVr for each region reference is reflected in the CL scale. Amyloid PET is interpreted by visual evaluation, but quantitative indices may also be used as a reference. Assume that quantitative indices change with elapsed time after radiopharmaceutical administration. In such cases, thresholds for discriminating between positive and negative results should be treated with caution [20].

Also, the quantitative indices are only based on pixel values measured in the region of interest. Therefore, it is possible that differences in imaging equipment and acquisition protocols, as well as image processing conditions (e.g., various corrections and noise reduction filters) may affect the results [21]. When quantitative indices are used as a reference in addition to visual evaluation in the interpretation of amyloid PET, it may be desirable to determine a threshold at one's institution for discriminating between positive and negative results.

**Fig. 9:**
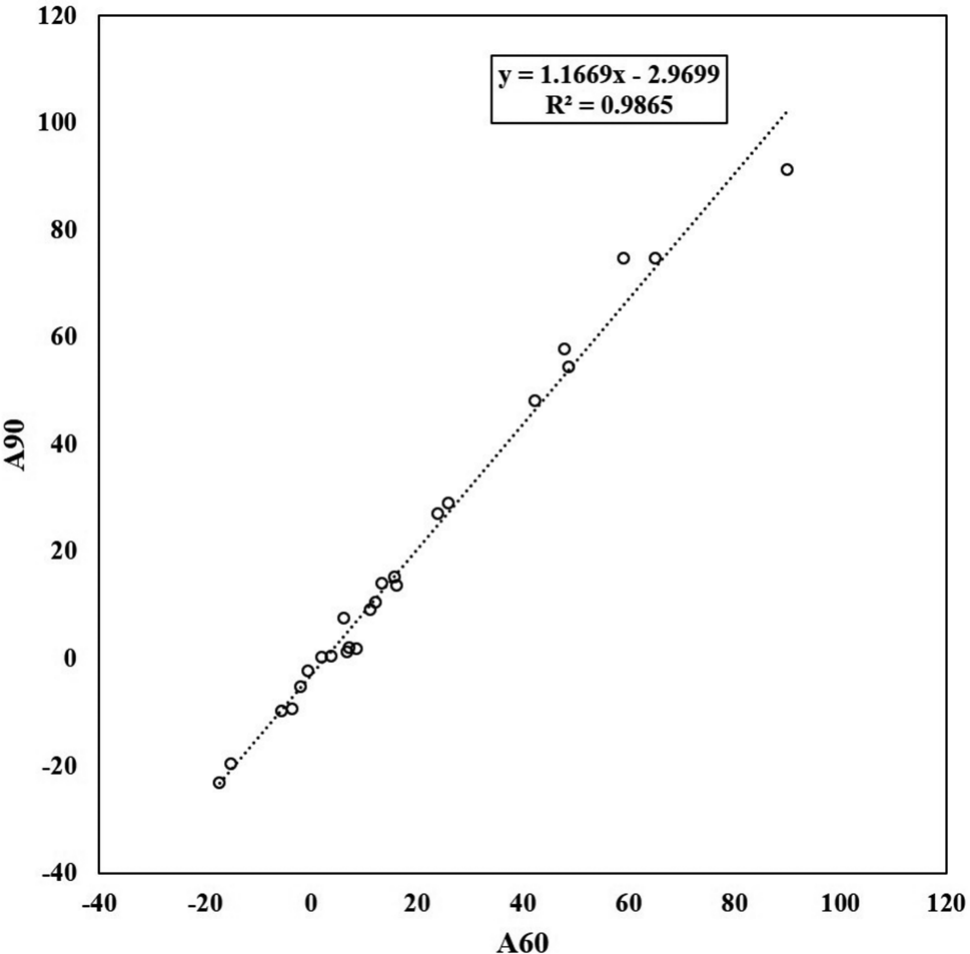
Scatter plot with CL scale for 60 and 90 min of waiting time

**Fig. 10:**
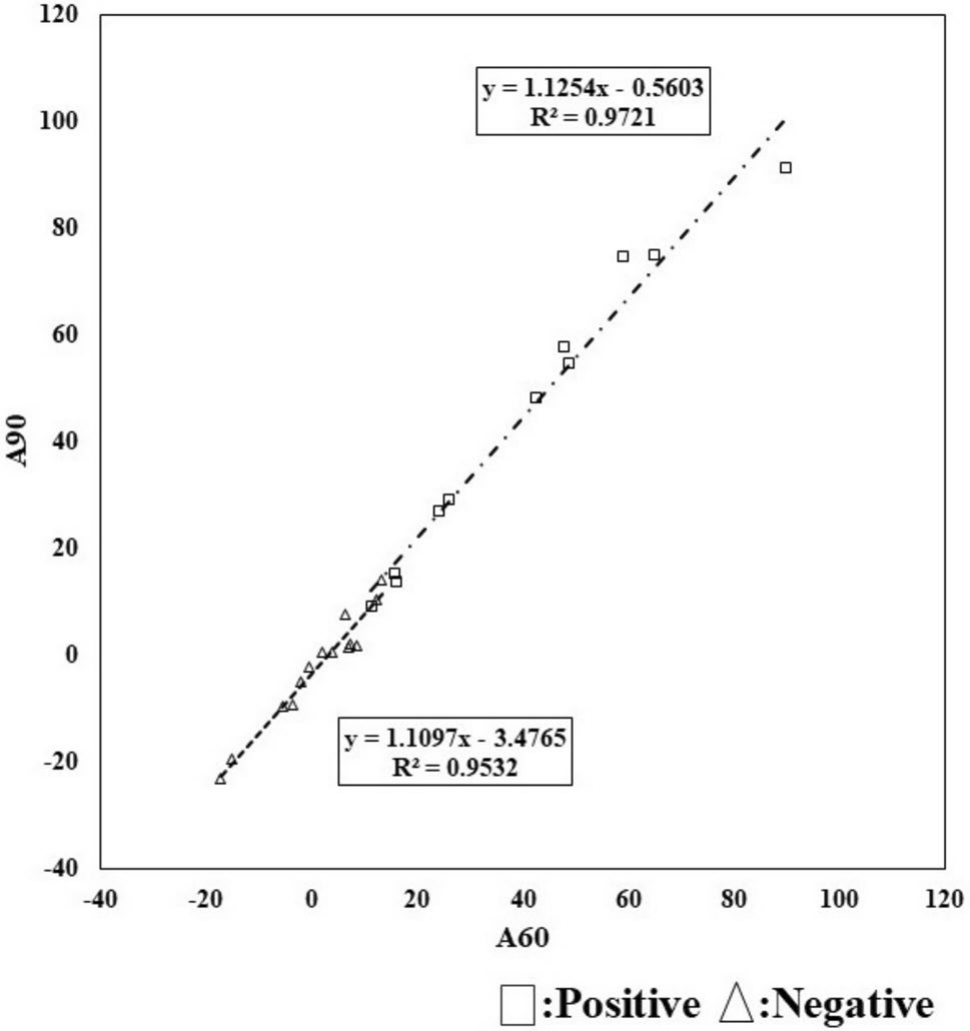
Scatter plots of the CL scale for 60 and 90 min waiting time in the negative and positive groups

As an experiment, we plotted the CL scale obtained from images with a waiting time of 60 min on the horizontal line and the CL scale obtained from images with a waiting time of 90 min on the vertical line which shows a linear correlation between the two. (Fig. 9). A similar linear correlation was also seen in the graph consisting of only 14 negative group and 11 positive group (Fig. 10).

This suggests that the effect of waiting time on the CL scale may be corrected by a linear equation. From the 25 cases in this study, we were able to derive the correlation formula:

$$A060{ }\left( {\text{after correction}} \right){ } = { }1.1669 \times A060{ }\left( {\text{before correction}} \right) - 2.9699$$

**Fig. 11:**
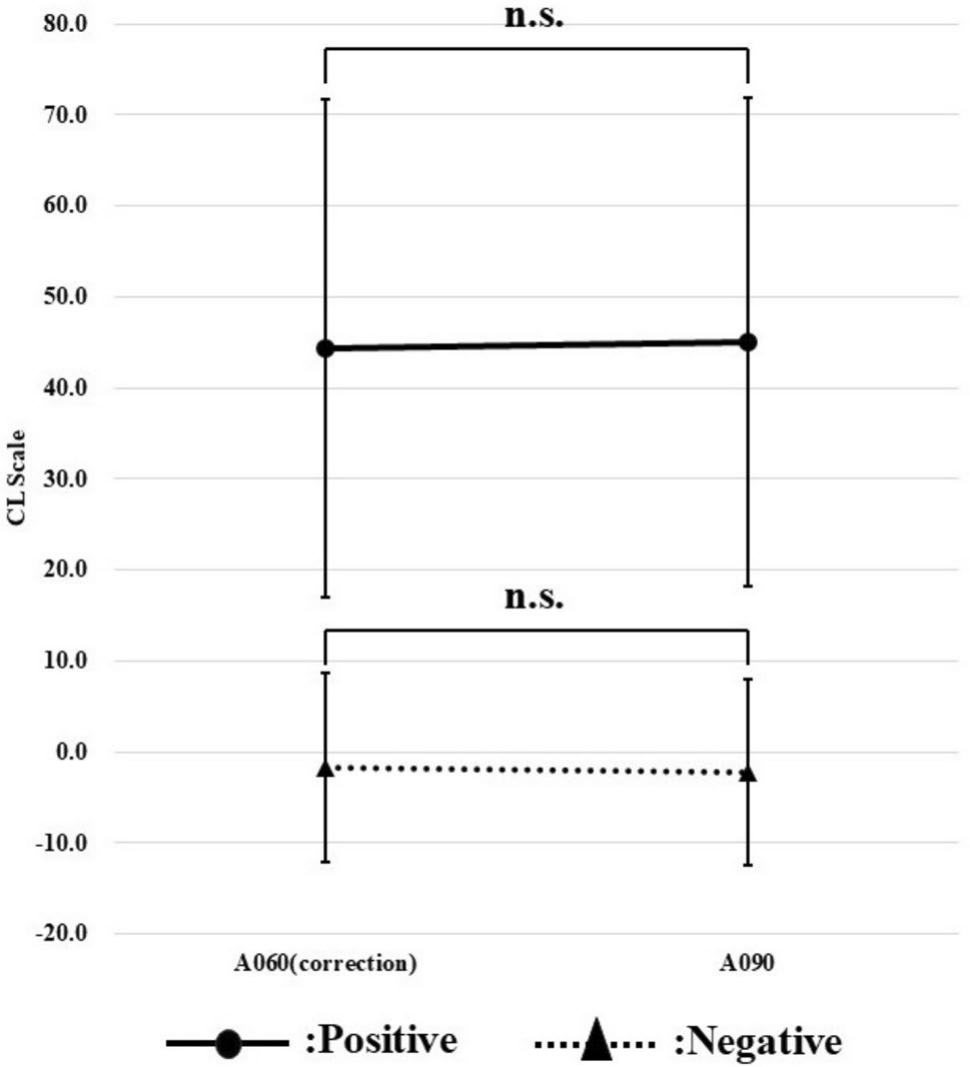
Comparison of CL scale (corrected value) obtained from a waiting time of 60 min with CL scale obtained from a waiting time of 90 min

Using this correlation equation, the CL scale obtained from images with a waiting time of 60 min was corrected and compared with the CL scale obtained from images with a waiting time of 90 min (Fig. 11). After correction using the correlation equation, there was no significant difference between the positive and negative groups for the CL scale at 60 and 90 min of waiting time. Therefore, when obtaining the CL scale, it was suggested that the difference due to waiting time could be corrected using the correlation equation.

However, it can be said that future discussion is needed regarding correction using correlation equations. Because, the correlation equation was obtained from a limited number of cases 25 subjects (positive group: 11, negative group: 14). In addition, in this study, anatomic standardization was performed using only PET images, so there is a limit to the accuracy of anatomic standardization. For example, this could be improved by calculating deformation parameters using MRI images of the same patient. Furthermore, because the results are based on PET images taken at our hospital, it is not yet clear how differences in PET equipment used and image processing conditions affect the results. Therefore, care still needs to be taken in handling the correlation equation.

## Conclusions

The present study revealed the relationship between the elapsed time since the administration of ^18^F-flutemetamol and the amount of radiopharmaceutical accumulation in the brain by pixel values within the region of interest (CL VOI). The effects on quantitative indices (SUVr and CL) were also confirmed.
